# Supplementary figures and images for: Antibiotic potentiation and inhibition of cross-resistance in pathogens associated with cystic fibrosis
Source: eLife. 2026 Apr 21;12:RP91082. doi: 10.7554/eLife.91082 (PMC13099141; doi:10.7554/eLife.91082)

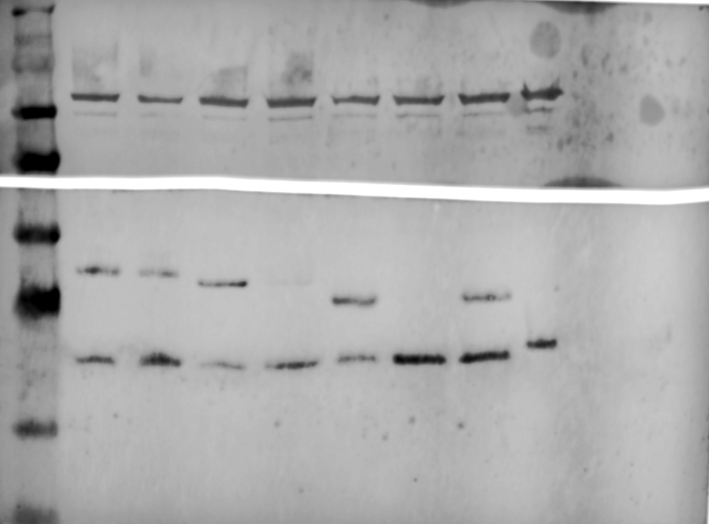

Supplement: Figure 2—source data 1. — ‘Left’, ‘Middle’, and ‘Right’ in the file names refer to the part of the immunoblot to the left, in-between, or to the right of the vertical black lines shown in the final figure, respectively. [file elife-91082-fig2-data1.zip › Figure 2-source data 1/Figure_2A_Left.png]

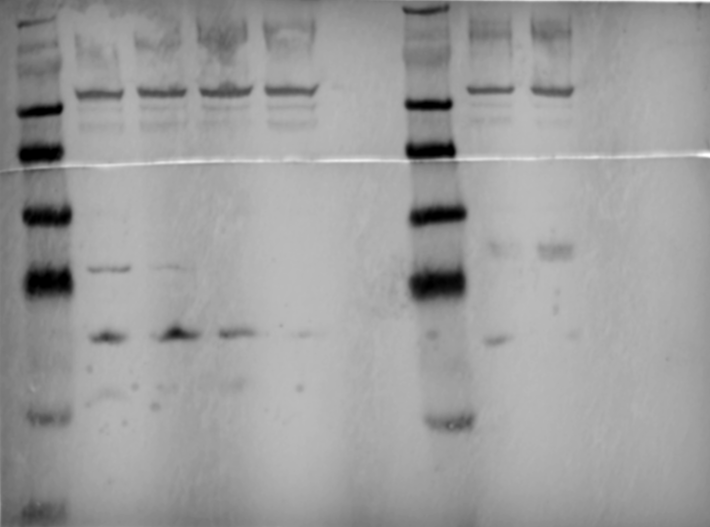

Supplement: Figure 2—source data 1. — ‘Left’, ‘Middle’, and ‘Right’ in the file names refer to the part of the immunoblot to the left, in-between, or to the right of the vertical black lines shown in the final figure, respectively. [file elife-91082-fig2-data1.zip › Figure 2-source data 1/Figure_2A_Middle.png]

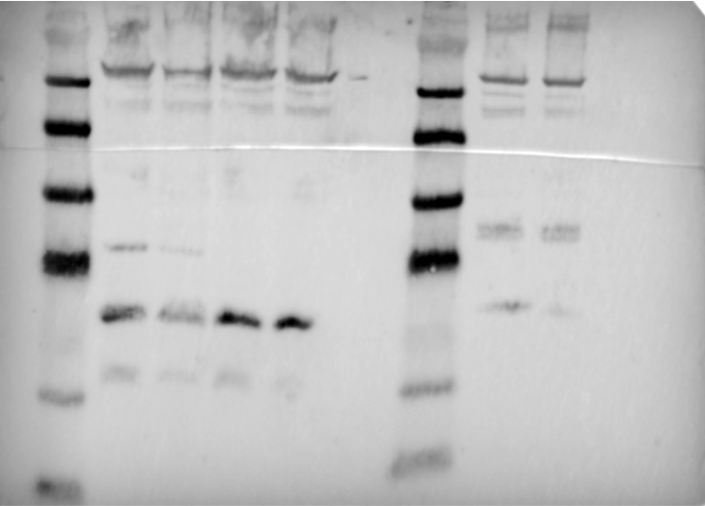

Supplement: Figure 2—source data 1. — ‘Left’, ‘Middle’, and ‘Right’ in the file names refer to the part of the immunoblot to the left, in-between, or to the right of the vertical black lines shown in the final figure, respectively. [file elife-91082-fig2-data1.zip › Figure 2-source data 1/Figure_2A_Right.png]

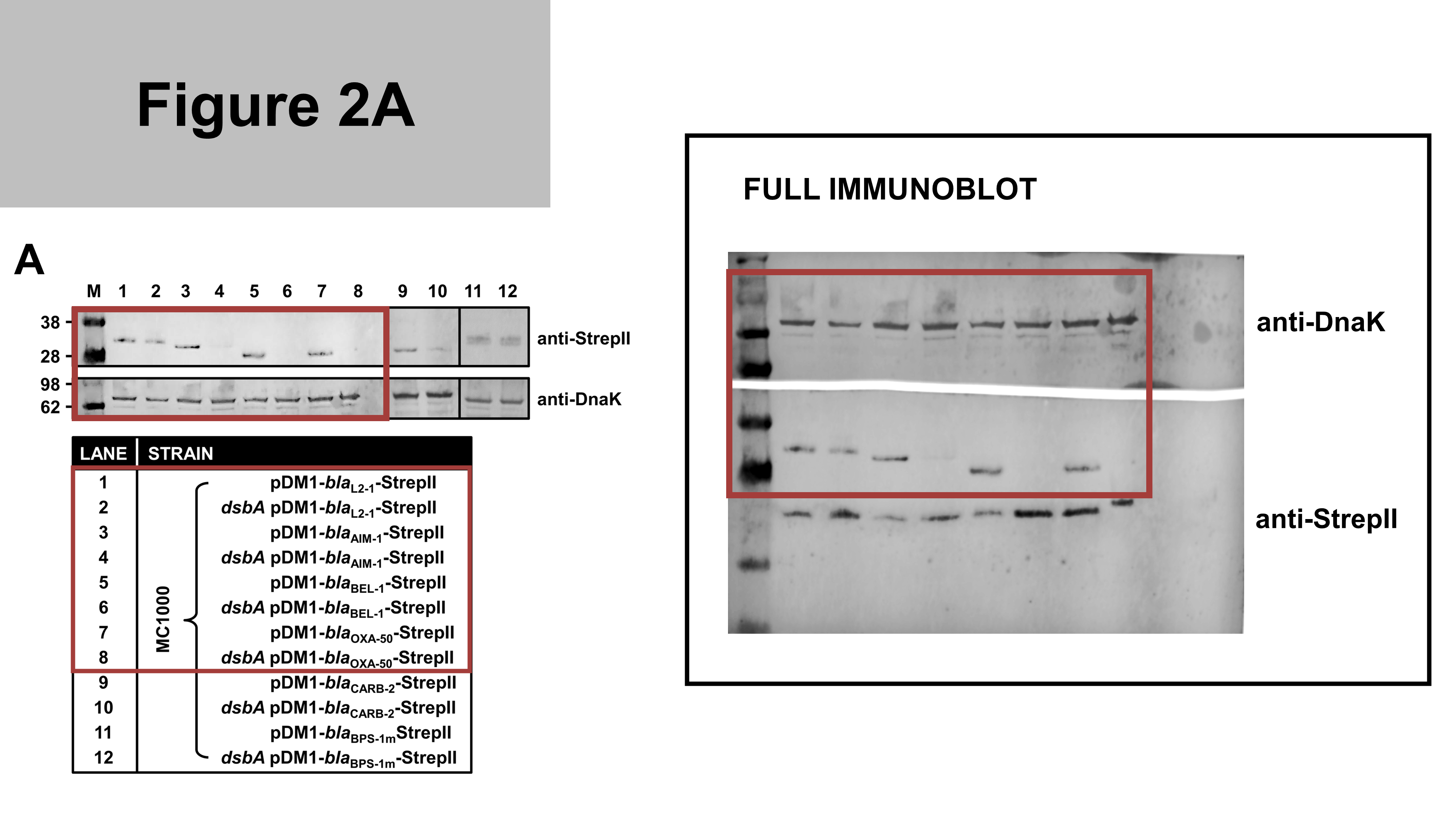

Supplement: Figure 2—source data 2. — The figure included in the paper is shown on the left and relevant bands used for each part of the figure are marked with red boxes on the uncropped immunoblots on the right. ‘Left’, ‘Middle’, and ‘Right’ in the file names refer to the part of the immunoblot to the left, in-between, or to the right of the vertical black lines shown in the final figure, respectively. [file elife-91082-fig2-data2.zip › Figure 2-source data 2/Figure_2A_Left.tif]

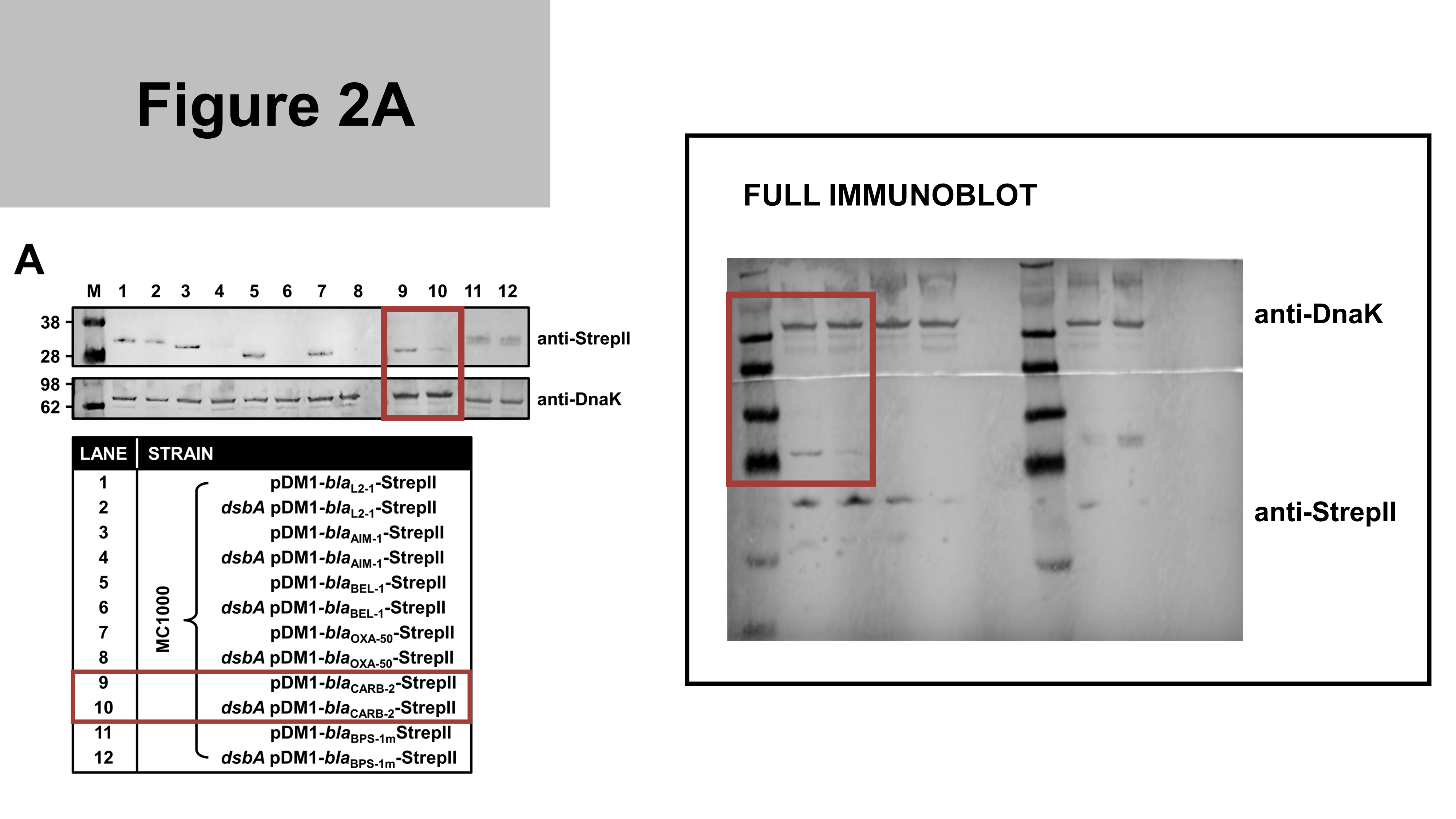

Supplement: Figure 2—source data 2. — The figure included in the paper is shown on the left and relevant bands used for each part of the figure are marked with red boxes on the uncropped immunoblots on the right. ‘Left’, ‘Middle’, and ‘Right’ in the file names refer to the part of the immunoblot to the left, in-between, or to the right of the vertical black lines shown in the final figure, respectively. [file elife-91082-fig2-data2.zip › Figure 2-source data 2/Figure_2A_Middle.tif]

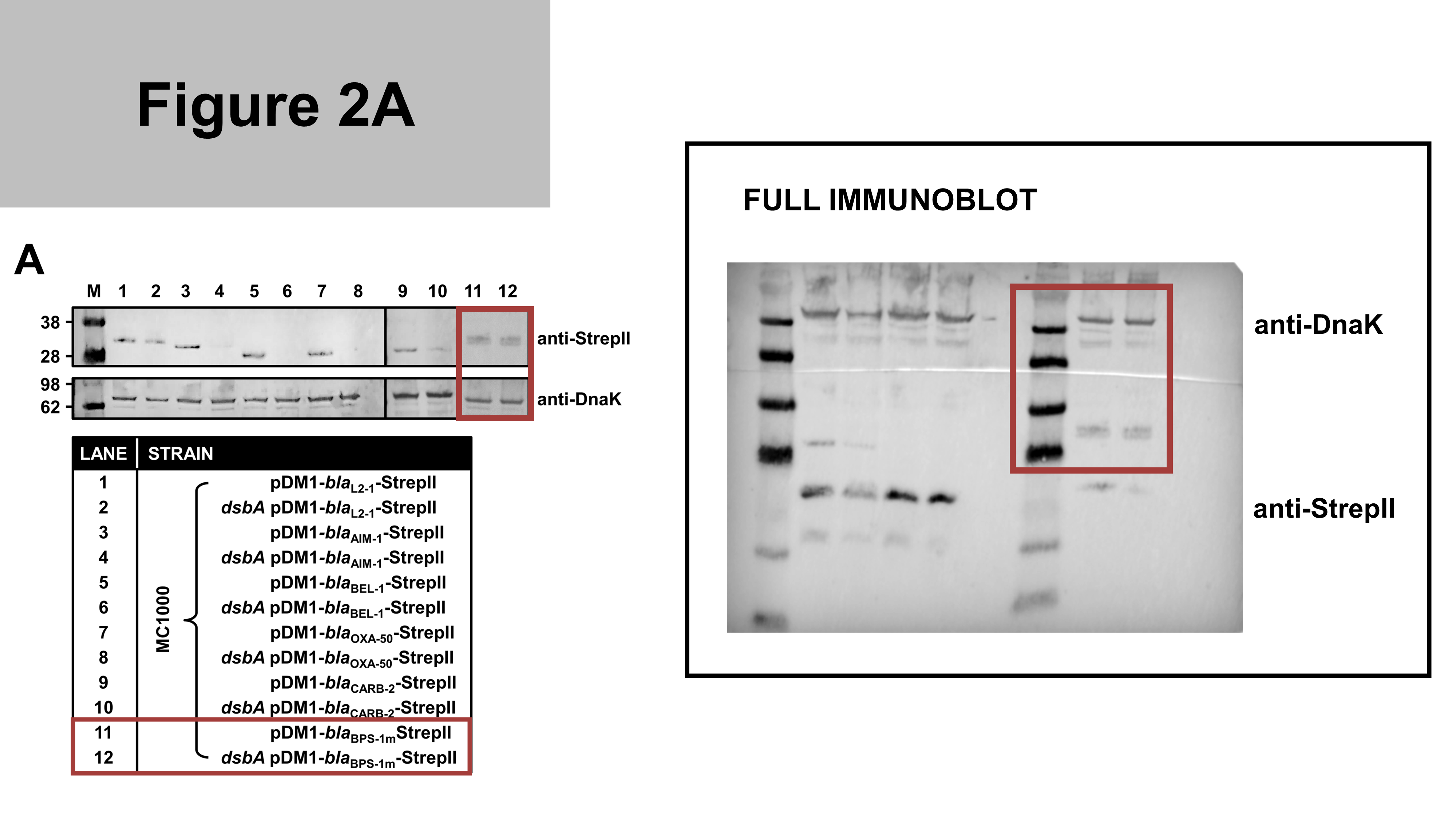

Supplement: Figure 2—source data 2. — The figure included in the paper is shown on the left and relevant bands used for each part of the figure are marked with red boxes on the uncropped immunoblots on the right. ‘Left’, ‘Middle’, and ‘Right’ in the file names refer to the part of the immunoblot to the left, in-between, or to the right of the vertical black lines shown in the final figure, respectively. [file elife-91082-fig2-data2.zip › Figure 2-source data 2/Figure_2A_Right.tif]

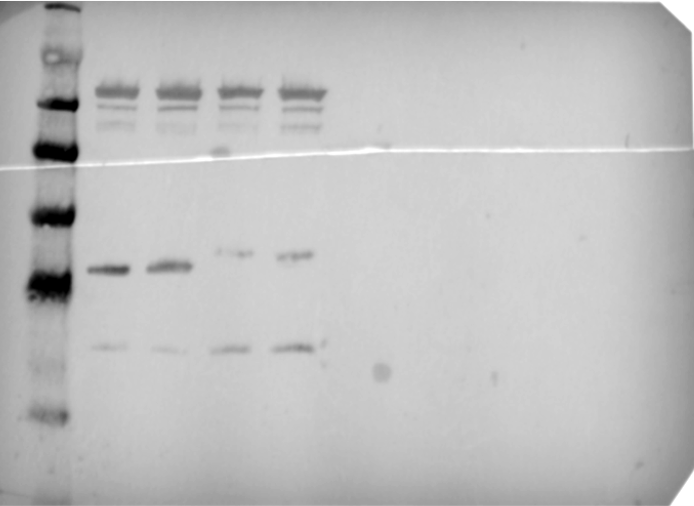

Supplement: Figure 5—figure supplement 1—source data 1. — ‘Left’ and ‘Right’ in the file names refer to the part of the immunoblot to the left or to the right of the vertical black line shown in the final figure, respectively. [file elife-91082-fig5-figsupp1-data1.zip › Figure 5 - figure supplement 1-source data 1/Figure_5-Figure_Supplement_1B_Left.png]

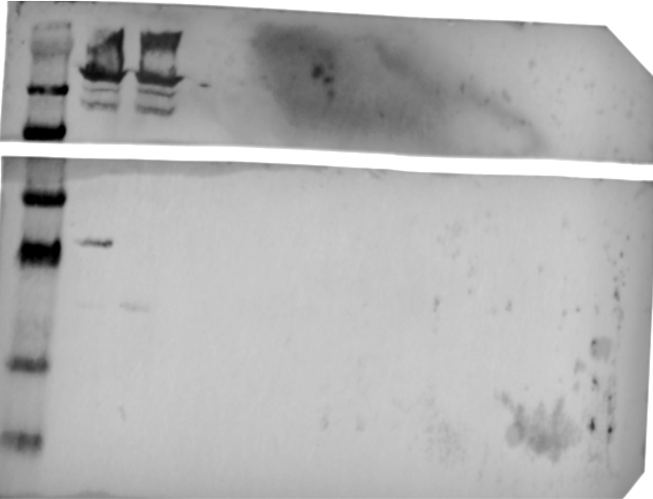

Supplement: Figure 5—figure supplement 1—source data 1. — ‘Left’ and ‘Right’ in the file names refer to the part of the immunoblot to the left or to the right of the vertical black line shown in the final figure, respectively. [file elife-91082-fig5-figsupp1-data1.zip › Figure 5 - figure supplement 1-source data 1/Figure_5-Figure_Supplement_1B_Right.png]

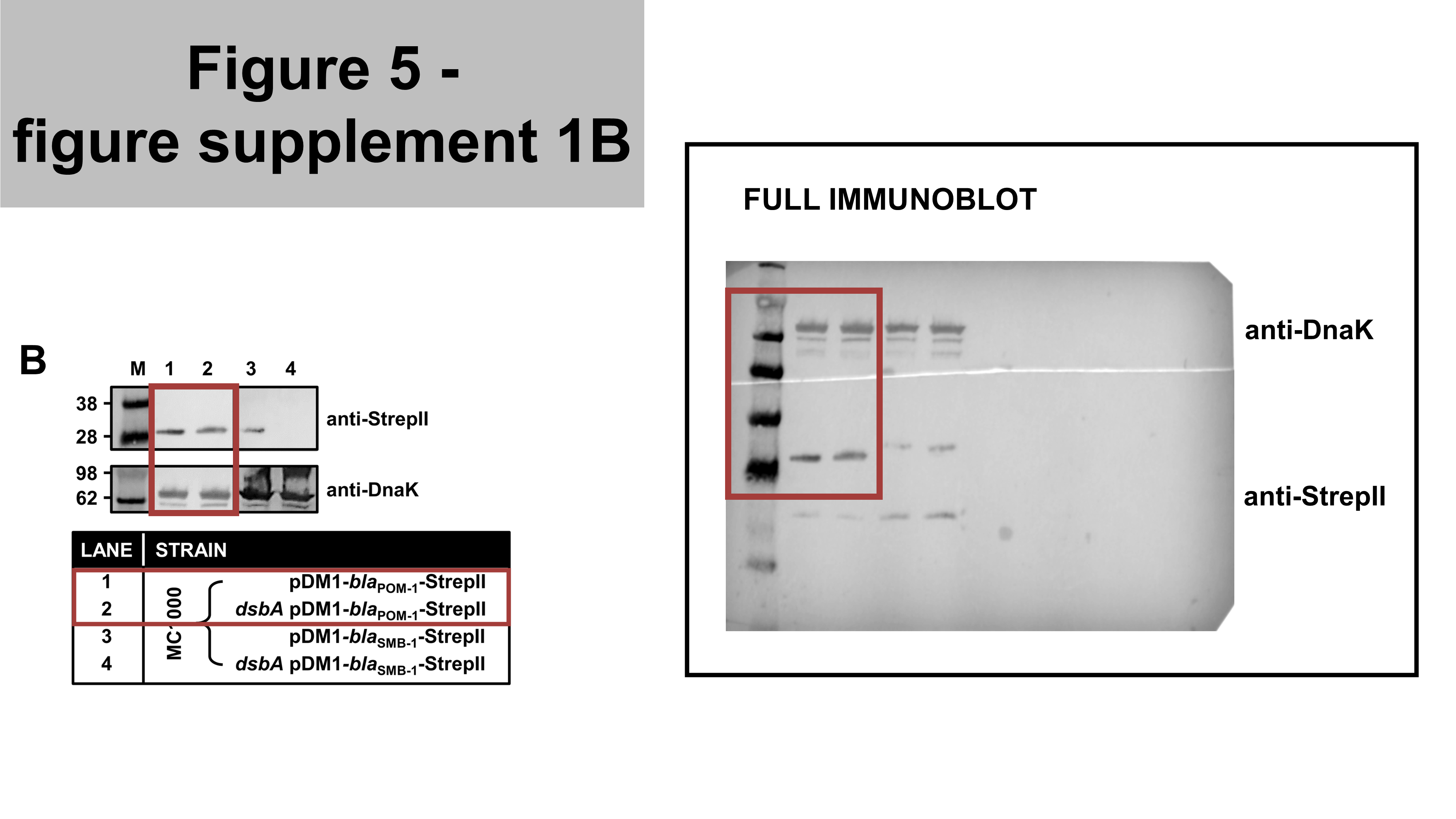

Supplement: Figure 5—figure supplement 1—source data 2. — The figure included in the paper is shown on the left and relevant bands used for each part of the figure are marked with red boxes on the uncropped immunoblots on the right. ‘Left’ and ‘Right’ in the file names refer to the part of the immunoblot to the left or to the right of the vertical black line shown in the final figure, respectively. [file elife-91082-fig5-figsupp1-data2.zip › Figure 5 - figure supplement 1-source data 2/Figure_5-Figure_supplement_1B_Left.tif]

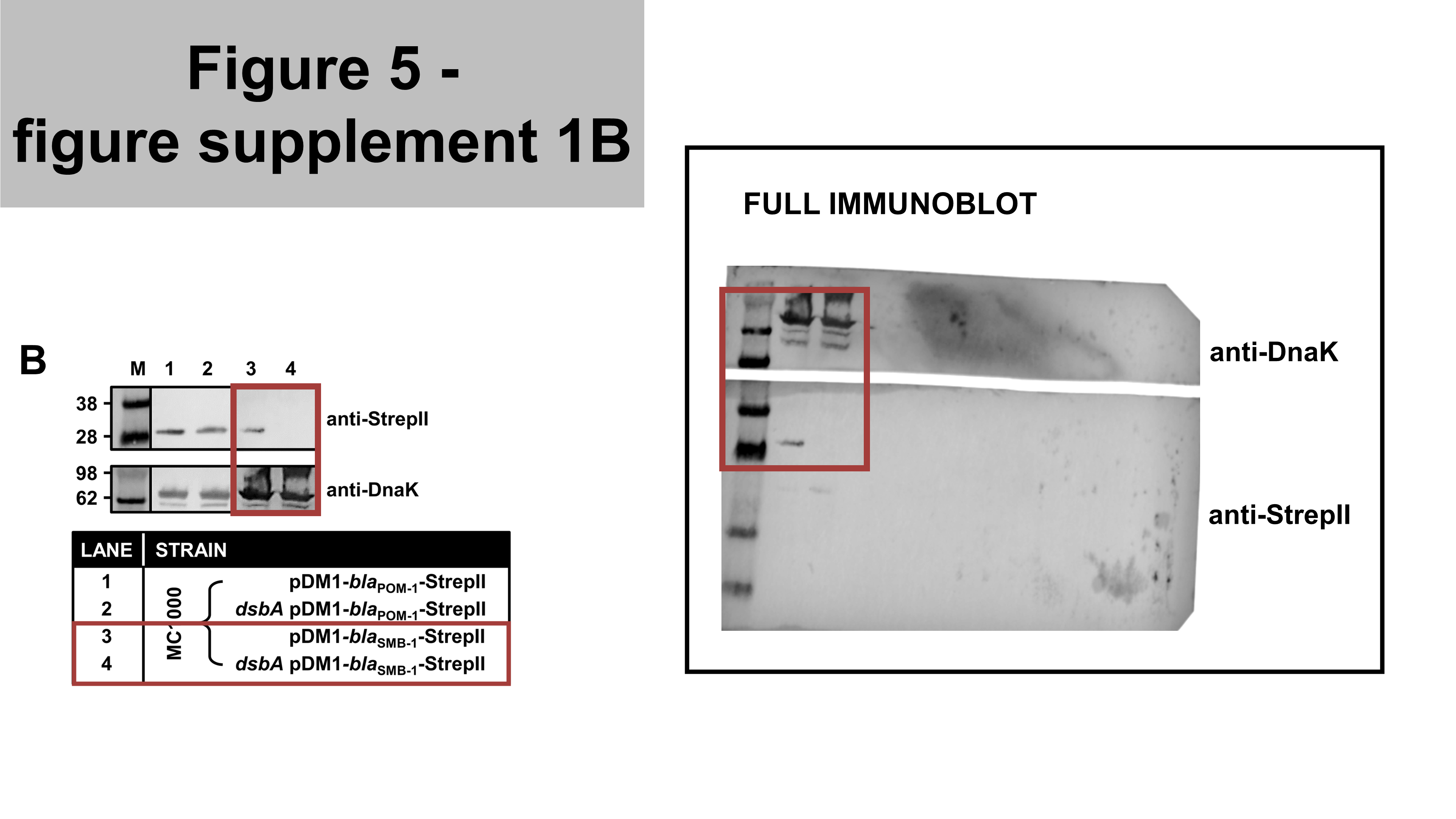

Supplement: Figure 5—figure supplement 1—source data 2. — The figure included in the paper is shown on the left and relevant bands used for each part of the figure are marked with red boxes on the uncropped immunoblots on the right. ‘Left’ and ‘Right’ in the file names refer to the part of the immunoblot to the left or to the right of the vertical black line shown in the final figure, respectively. [file elife-91082-fig5-figsupp1-data2.zip › Figure 5 - figure supplement 1-source data 2/Figure_5-Figure_supplement_1B_Right.tif]
