# Supplementary material for: Antibiotic potentiation and inhibition of cross-resistance in pathogens associated with cystic fibrosis
Source: eLife. 2026 Apr 21;12:RP91082. doi: 10.7554/eLife.91082 (PMC13099141; doi:10.7554/eLife.91082)

# IMMUNOBLOTS

# Figure 2A

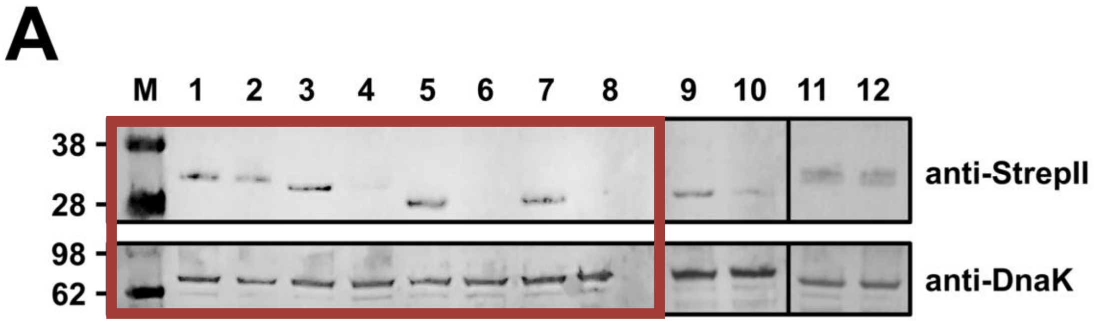


| LANE | STRAIN | |
|---|---|---|
| 1 | MC1000 | pDM1-$bla_{L2-1}$-StrepII |
| 2 | | *dsbA* pDM1-$bla_{L2-1}$-StrepII |
| 3 | | pDM1-$bla_{AIM-1}$-StrepII |
| 4 | | *dsbA* pDM1-$bla_{AIM-1}$-StrepII |
| 5 | | pDM1-$bla_{BEL-1}$-StrepII |
| 6 | | *dsbA* pDM1-$bla_{BEL-1}$-StrepII |
| 7 | | pDM1-$bla_{OXA-50}$-StrepII |
| 8 | | *dsbA* pDM1-$bla_{OXA-50}$-StrepII |
| 9 | | pDM1-$bla_{CARB-2}$-StrepII |
| 10 | | *dsbA* pDM1-$bla_{CARB-2}$-StrepII |
| 11 | | pDM1-$bla_{BPS-1m}$StrepII |
| 12 | | *dsbA* pDM1-$bla_{BPS-1m}$-StrepII |

**FULL IMMUNOBLOT**

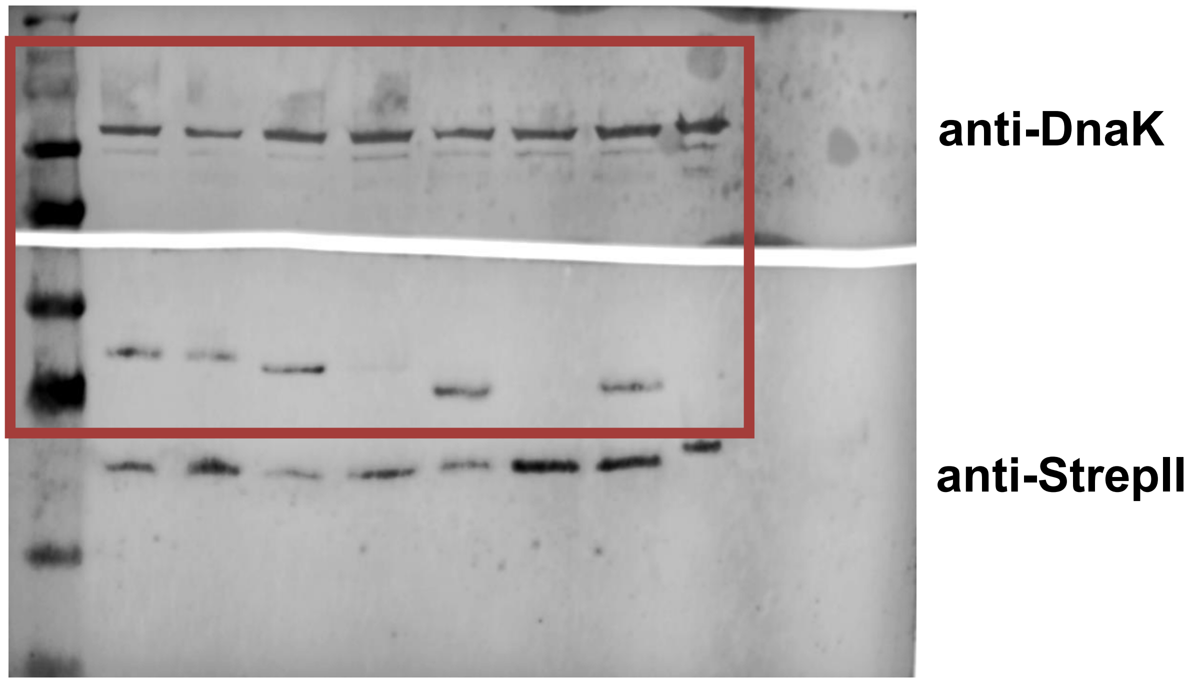

# Figure 2A

**A**

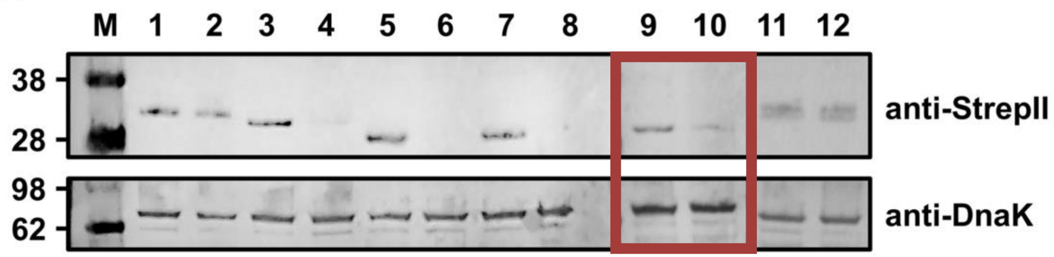


| LANE | STRAIN | |
|---|---|---|
| 1 | MC1000 | pDM1-*bla*$_{L2-1}$-StrepII |
| 2 | | *dsbA* pDM1-*bla*$_{L2-1}$-StrepII |
| 3 | | pDM1-*bla*$_{AIM-1}$-StrepII |
| 4 | | *dsbA* pDM1-*bla*$_{AIM-1}$-StrepII |
| 5 | | pDM1-*bla*$_{BEL-1}$-StrepII |
| 6 | | *dsbA* pDM1-*bla*$_{BEL-1}$-StrepII |
| 7 | | pDM1-*bla*$_{OXA-50}$-StrepII |
| 8 | | *dsbA* pDM1-*bla*$_{OXA-50}$-StrepII |
| 9 | | pDM1-*bla*$_{CARB-2}$-StrepII |
| 10 | | *dsbA* pDM1-*bla*$_{CARB-2}$-StrepII |
| 11 | | pDM1-*bla*$_{BPS-1m}$StrepII |
| 12 | | *dsbA* pDM1-*bla*$_{BPS-1m}$-StrepII |

## FULL IMMUNOBLOT

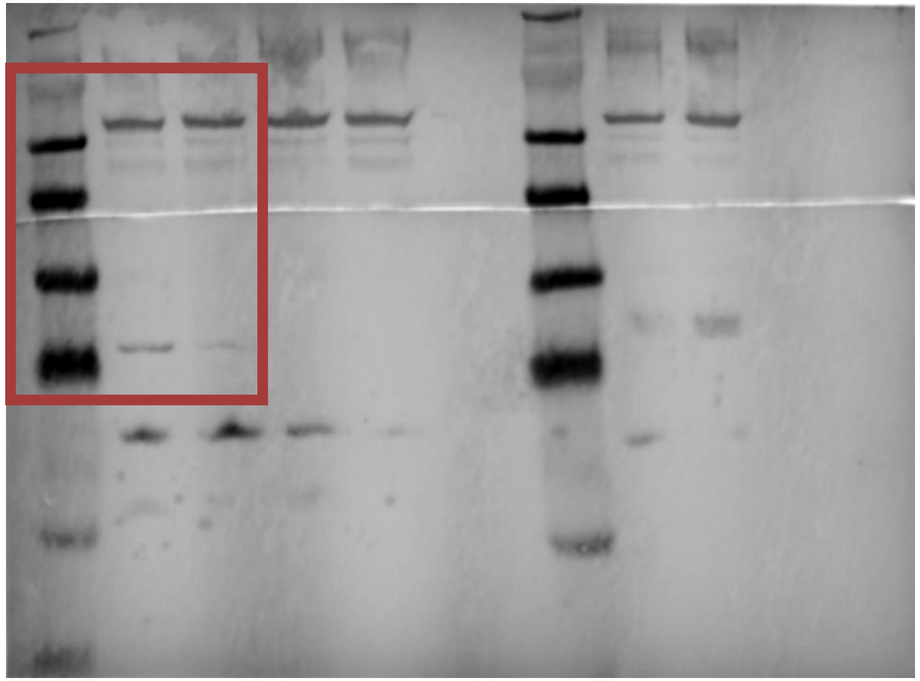

# Figure 2A

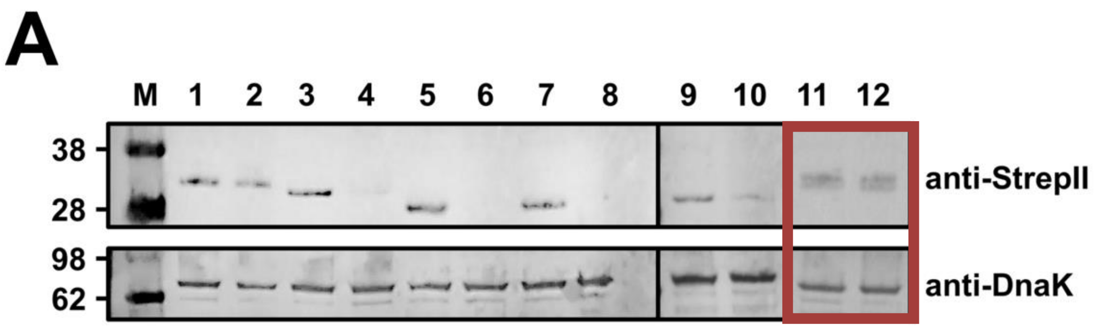


| LANE | STRAIN | |
|---|---|---|
| 1 | MC1000 | pDM1-$bla_{L2-1}$-StrepII |
| 2 | | *dsbA* pDM1-$bla_{L2-1}$-StrepII |
| 3 | | pDM1-$bla_{AIM-1}$-StrepII |
| 4 | | *dsbA* pDM1-$bla_{AIM-1}$-StrepII |
| 5 | | pDM1-$bla_{BEL-1}$-StrepII |
| 6 | | *dsbA* pDM1-$bla_{BEL-1}$-StrepII |
| 7 | | pDM1-$bla_{OXA-50}$-StrepII |
| 8 | | *dsbA* pDM1-$bla_{OXA-50}$-StrepII |
| 9 | | pDM1-$bla_{CARB-2}$-StrepII |
| 10 | | *dsbA* pDM1-$bla_{CARB-2}$-StrepII |
| 11 | | pDM1-$bla_{BPS-1m}$StrepII |
| 12 | | *dsbA* pDM1-$bla_{BPS-1m}$-StrepII |

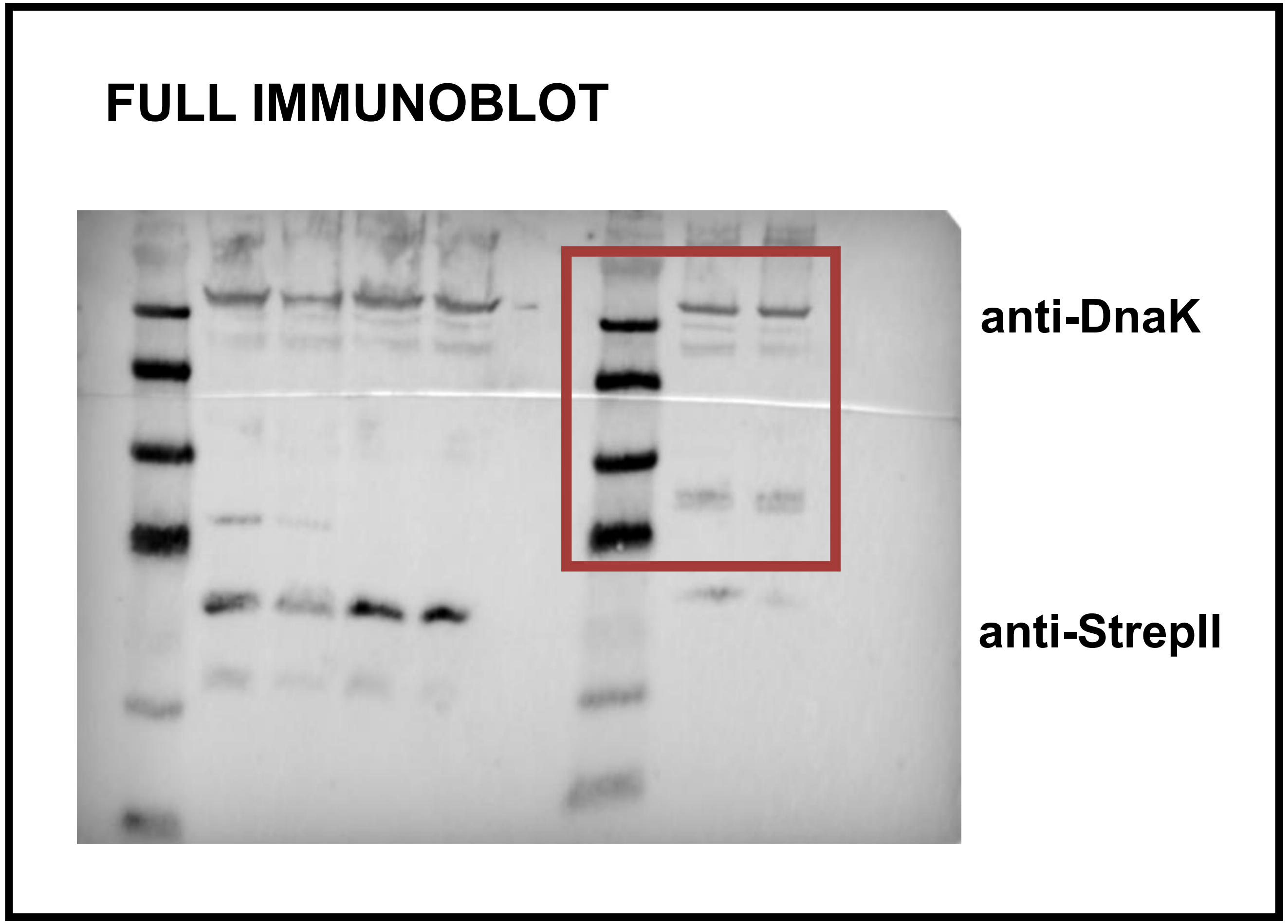

# Figure 5 - figure supplement 1B

**B**

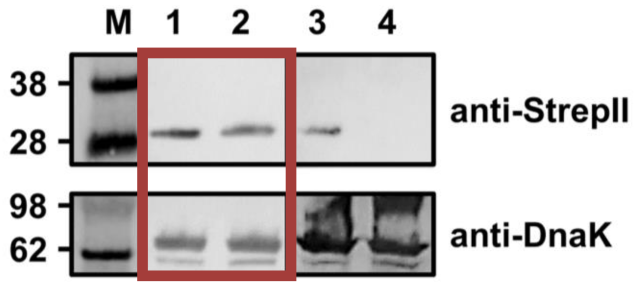


| LANE | STRAIN | |
|---|---|---|
| 1 | MC[illegible]000 | pDM1-$bla_{POM-1}$-StrepII |
| 2 | | *dsbA* pDM1-$bla_{POM-1}$-StrepII |
| 3 | | pDM1-$bla_{SMB-1}$-StrepII |
| 4 | | *dsbA* pDM1-$bla_{SMB-1}$-StrepII |

**FULL IMMUNOBLOT**

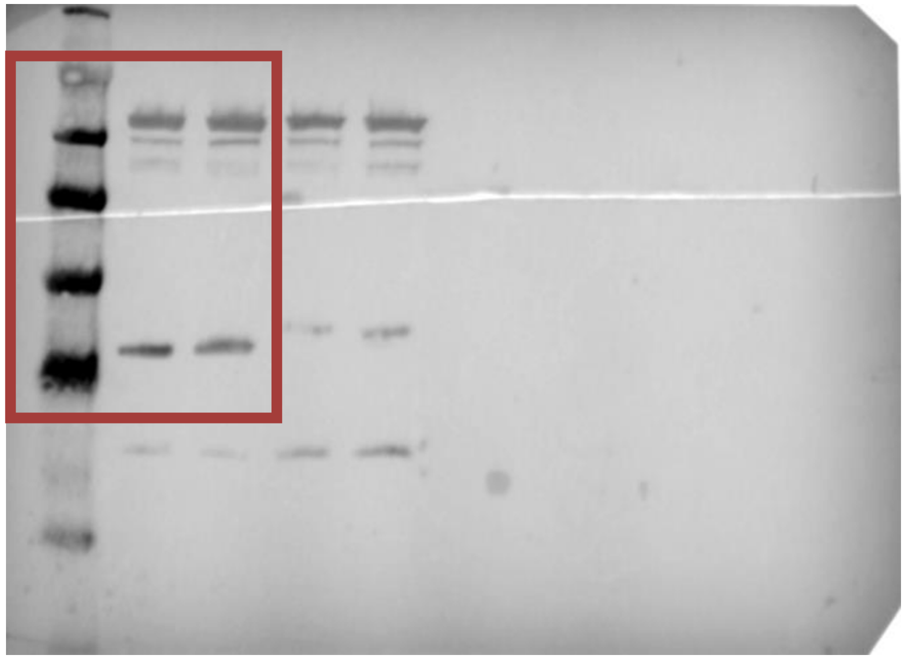

# Figure 5 - figure supplement 1B

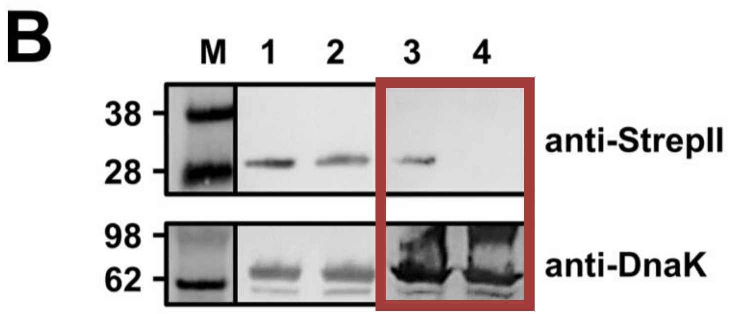


| LANE | STRAIN | |
|---|---|---|
| 1 | MC[illegible]000 | pDM1-$bla_{POM-1}$-StrepII |
| 2 | | *dsbA* pDM1-$bla_{POM-1}$-StrepII |
| 3 | | pDM1-$bla_{SMB-1}$-StrepII |
| 4 | | *dsbA* pDM1-$bla_{SMB-1}$-StrepII |

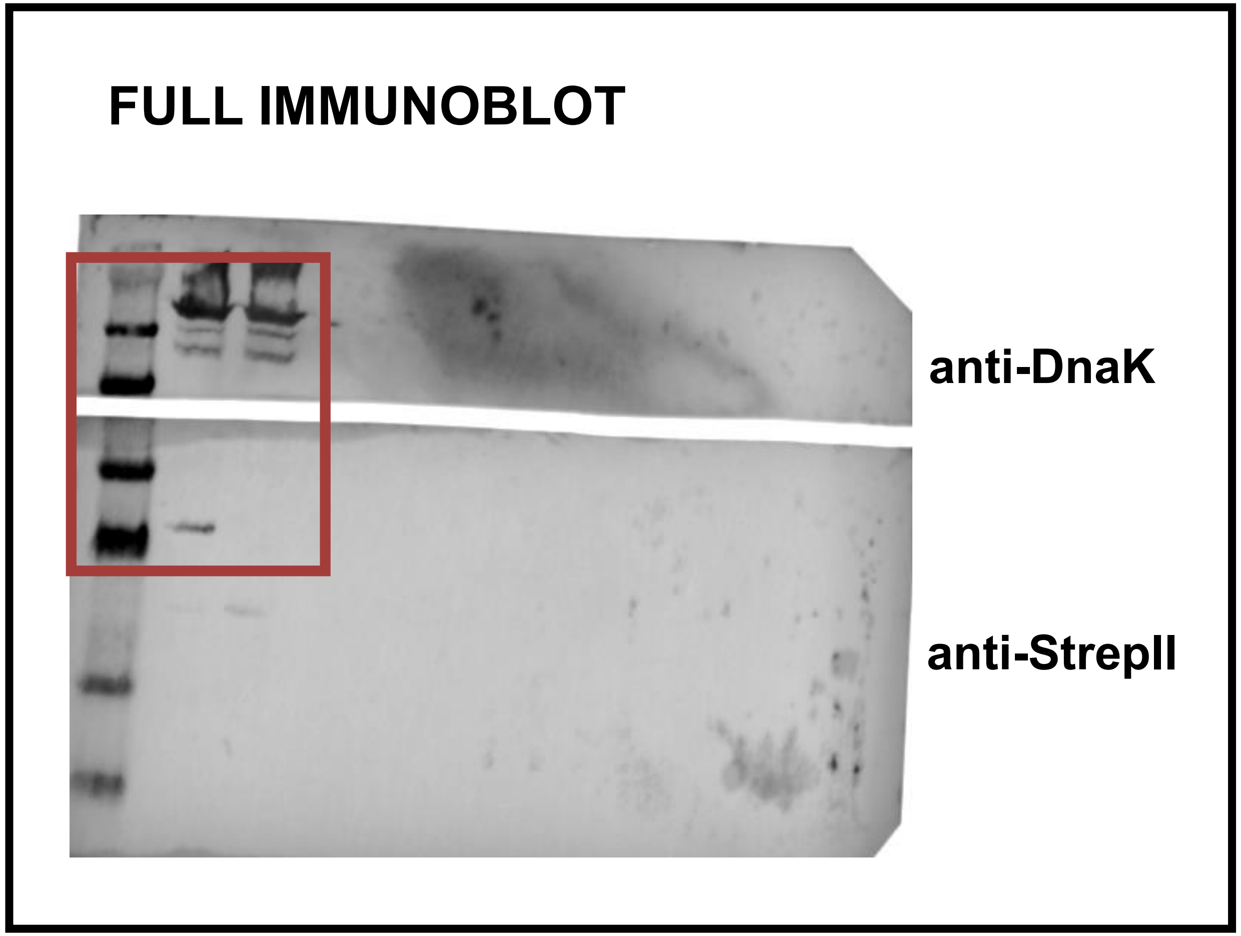

# SDS PAGE GELS

# Figure 2A

SDS-PAGE analysis of immunoblot samples for total protein content

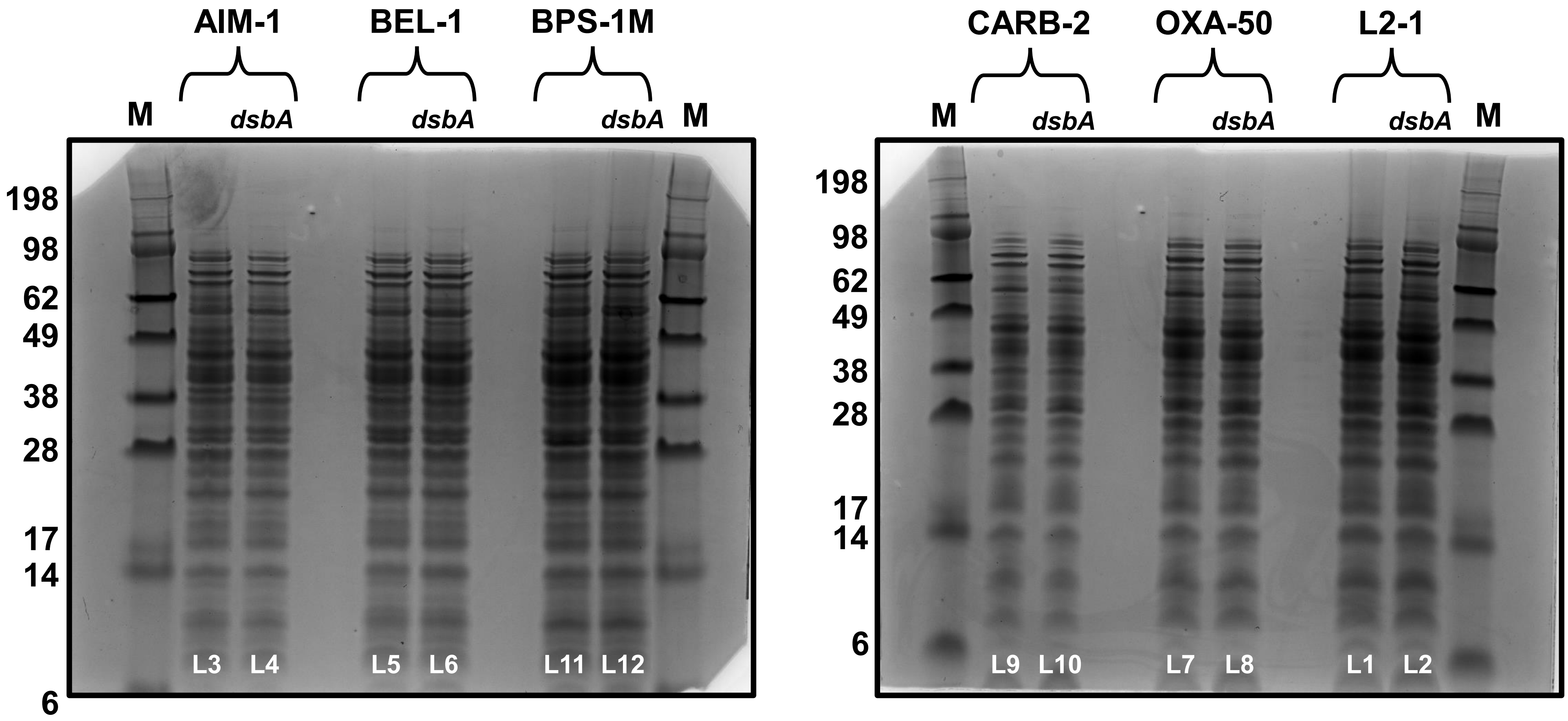

# Figure 5 - figure supplement 1B

SDS-PAGE analysis of immunoblot samples for total protein content

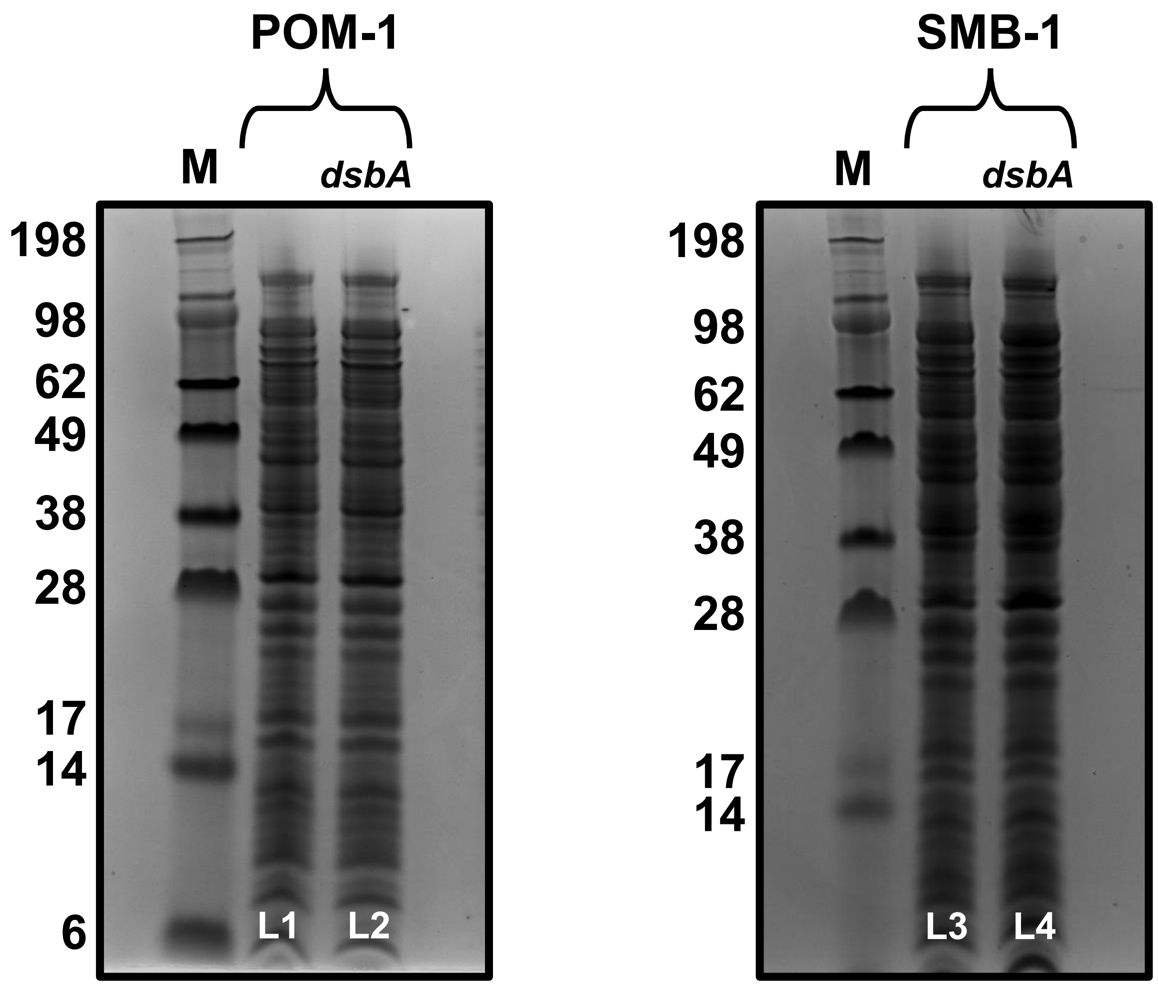

Supplement: Supplementary file 3. — (Pages 1–6). Full immunoblots for Figure 2A, Figure 5—figure supplement 1B. On the left of each page, the relevant figure panel is shown and the lanes in question are marked with red outline. On the right of each page, the full immunoblot is displayed with the corresponding area also marked with red outline. (Pages 7–9) SDS PAGE analysis of the immunoblot samples for total protein content. In each page, the immunoblot in question is indicated (by “Figure 2A” or “Figure 5—figure supplement 1B”) and lanes are marked accordingly to identify the immunoblot lane that they correspond to (see white labels at the bottom of the gel). [file elife-91082-supp3.pdf]
