# Supplementary material for: Antibiotic potentiation and inhibition of cross-resistance in pathogens associated with cystic fibrosis
Source: eLife. 2026 Apr 21;12:RP91082. doi: 10.7554/eLife.91082 (PMC13099141; doi:10.7554/eLife.91082)
Supplement: Supplementary file 5. — 1H-NMR and LCMS spectra of 4,5-dibromo-2-(2-chlorobenzyl)pyridazin-3(2 H)-one (compound 36) demonstrating the correctness and purity of the synthesized compound by Bioduro-Sundia. [file elife-91082-supp5.pdf]

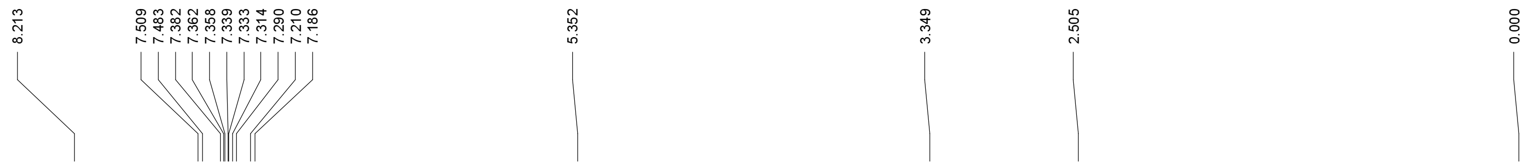

Varian Mercury Plus: 300 MHz
Solvent: DMSO
Y1359-08443-024

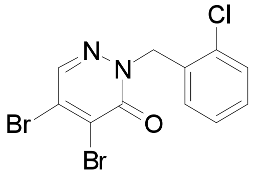

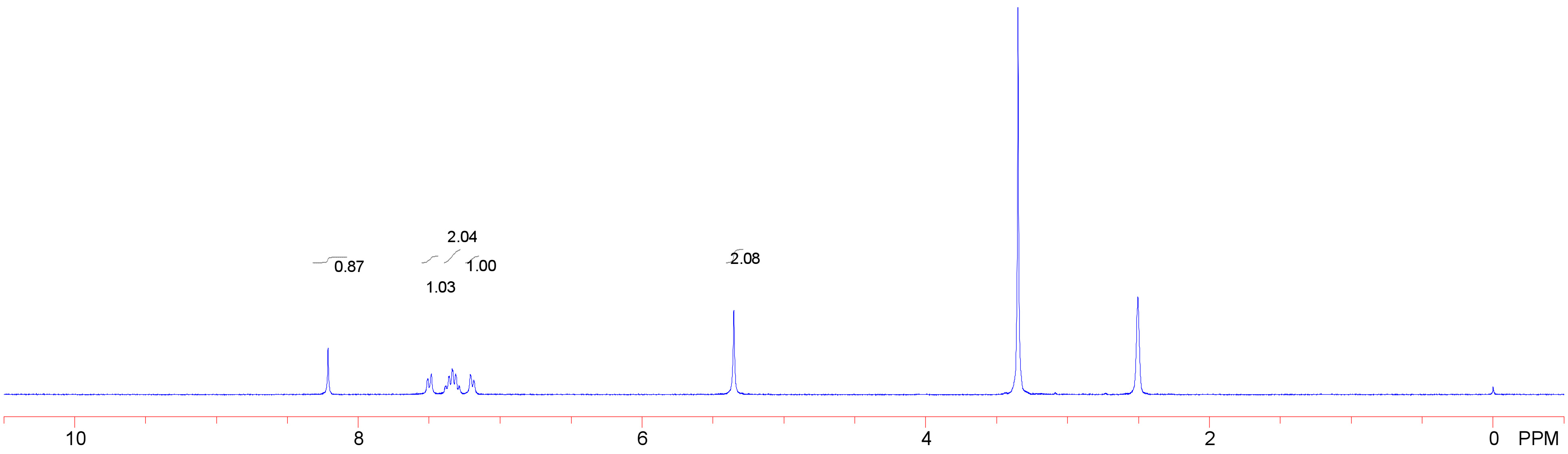

Sundia Meditech Co.Ltd

QC Report

388 Jialilue Road
Zhangjiang Hightech Park
Shanghai, China
Post code : 201203
Tel : 8621-51098642
Fax : 8621-51903505

Sample Name	: Y1359-08443-024			
Data File	: D:\DATA-LCMS-3\2015\02\28\2015-02-28--1\Y1359-08443-02->			
Injection Date	: Sat, 28. Feb. 2015	Inj. Vol.	:	2ul
Acq Operator	: Shao Yan	Location	:	Vial 4
Acq. Method	: D:\DATA-LCMS-3\2015\02\28\2015-02-28--1\P-30-95.M			
Sample Info.:				
Methods Info:	column:C18;column size:4.6*50mm ;mobile phase:B(ACN), A(0.02%NH4Ac); gradient(B%):as Acq.Method above			

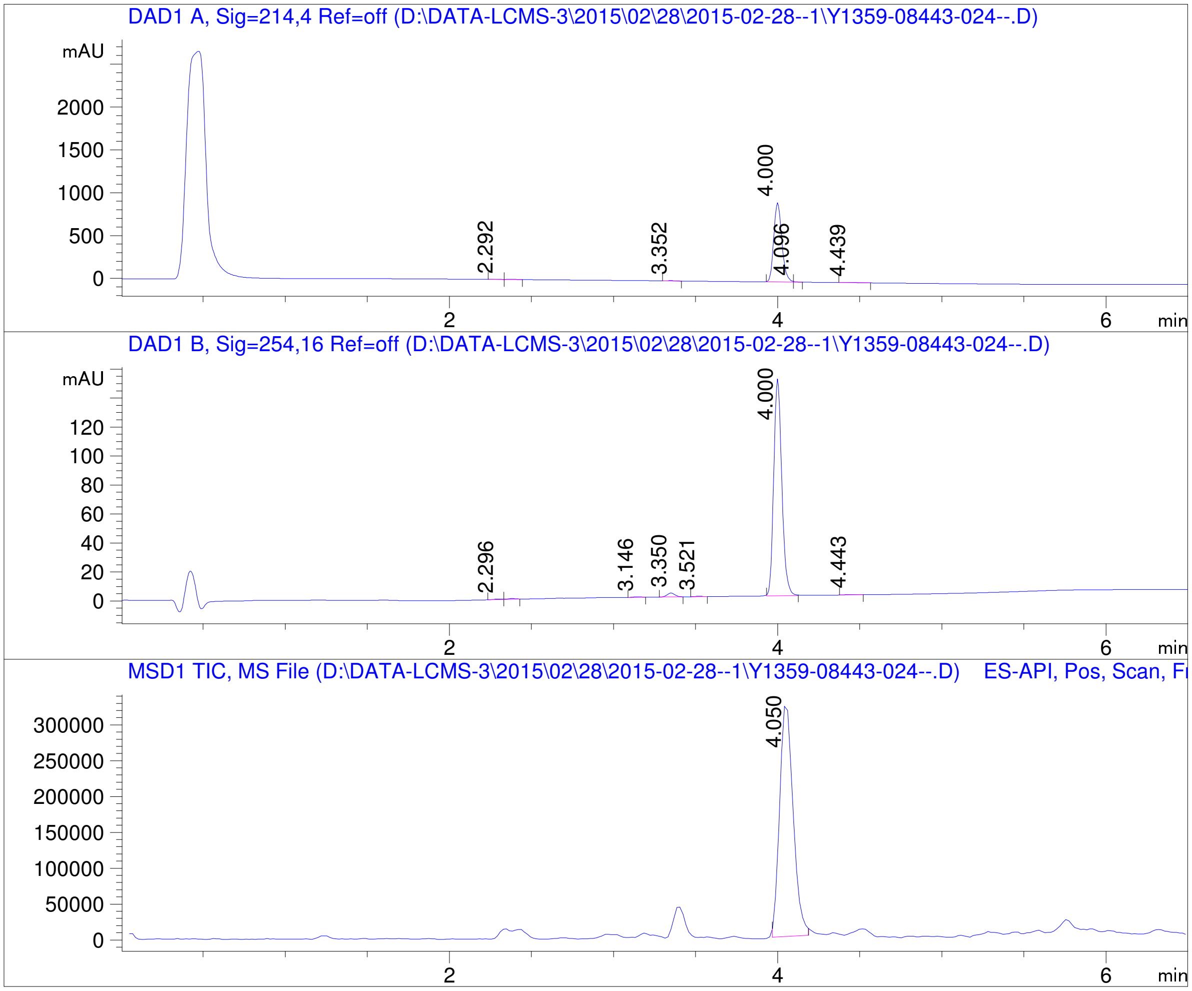

===

Integration Results

===

Signal 1: DAD1 A, Sig=214,4 Ref=off

#	R.T.	Type	Height	Height%	Width	Area	Area %
1	2.292	MF	1.350	0.142	0.062	5.039	0.156
2	2.379	FM	4.603	0.485	0.056	15.501	0.481
3	3.352	MF	4.205	0.443	0.055	13.969	0.434
4	4.000	FM	928.564	97.871	0.057	3164.489	98.265
5	4.096	FM	7.268	0.766	0.015	6.688	0.208
6	4.439	MF	2.777	0.293	0.088	14.675	0.456

#	R.T.	Type	Height	Height%	Width	Area	Area %

Signal 2: DAD1 B, Sig=254,16 Ref=off

#	R.T.	Type	Height	Height%	Width	Area	Area %
1	2.296	MF	0.367	0.237	0.054	1.187	0.225
2	2.384	FM	0.530	0.343	0.057	1.803	0.341
3	3.146	MM	0.432	0.280	0.054	1.411	0.267
4	3.350	MF	2.661	1.723	0.054	8.696	1.646
5	3.521	FM	0.297	0.192	0.060	1.076	0.204
6	4.000	BB	149.820	97.008	0.054	512.694	97.043
7	4.443	MM	0.334	0.216	0.072	1.450	0.274

Signal 3: MSD1 TIC, MS File

#	R.T.	Type	Height	Height%	Width	Area	Area %
1	4.050	FM	330220.750	100.000	0.093	1.846e+006	100.000

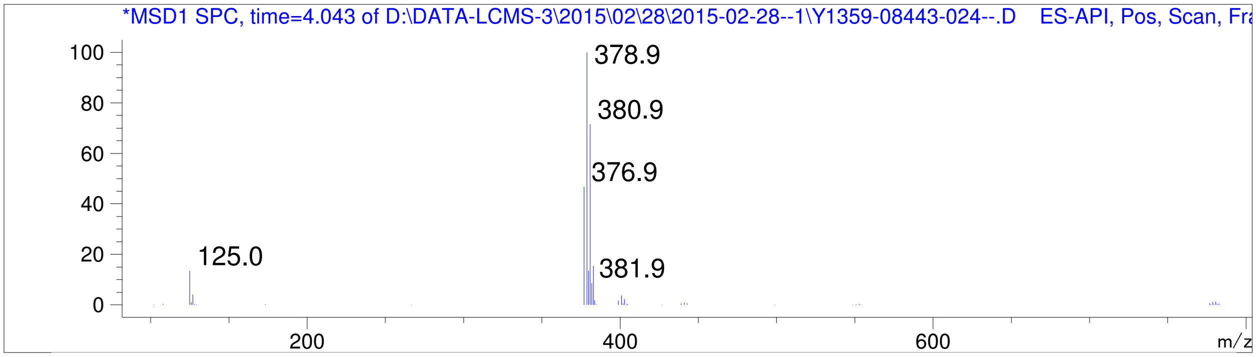

*** End of Report ***
