# Supplementary material for: Antibiotic potentiation and inhibition of cross-resistance in pathogens associated with cystic fibrosis
Source: eLife. 2026 Apr 21;12:RP91082. doi: 10.7554/eLife.91082 (PMC13099141; doi:10.7554/eLife.91082)
Supplement: Supplementary file 6. [file elife-91082-supp6.docx]

**SUPPLEMENTARY FILE FOR**

**Antibiotic potentiation and inhibition of cross-resistance in pathogens associated with cystic fibrosis**

Nikol Kadeřábková^1,3,†^, R. Christopher D. Furniss^3,†^, Evgenia Maslova^4^, Kathryn E. Potter^1^, Lara Eisaiankhongi^4^, Patricia Bernal^5^, Alain Filloux^3,6,7,8^, Cristina Landeta^9^, Diego Gonzalez^10^, Ronan R. McCarthy^4^, Despoina A.I. Mavridou^1,2^**^*^**

^1^Department of Molecular Biosciences, The University of Texas at Austin, Austin, 78712, Texas, USA

^2^John Ring LaMontagne Center for Infectious Diseases, The University of Texas at Austin, Austin, 78712, Texas, USA

^3^Centre for Bacterial Resistance Biology, Department of Life Sciences, Imperial College London, London, SW7 2AZ, UK

^4^Division of Biosciences, Department of Life Sciences, College of Health and Life Sciences,

Brunel University London, Uxbridge, UB8 3PH, UK

^5^Departamento de Microbiología, Facultad de Biología, Universidad de Sevilla, Seville, 41012, Spain

^6^Singapore Centre for Environmental Life Sciences Engineering, Nanyang Technological University, 637551, Singapore

^7^School of Biological Sciences, Nanyang Technological University, 639798, Singapore

^8^Lee Kon Chian School of Medicine, Nanyang Technological University, 636921, Singapore

^9^Department of Biology, Indiana University, Bloomington, Indiana, 47405, USA.

^10^Laboratoire de Microbiologie, Institut de Biologie, Université de Neuchâtel, Neuchâtel, 2000, Switzerland

**^*^**Correspondence: despoina.mavridou@austin.utexas.edu

^†^These authors have contributed equally to this work

**This PDF file includes:**

Supplementary Tables 1 to 5

Supplementary references

**SUPPLEMENTARY TABLES**

**Supplementary Table 1.** Overview of the β-lactamase enzymes investigated in this study. All tested enzymes belong to distinct phylogenetic clusters (see Supplementary File 1), with the exception of BPS-1m and BPS-6. The “Cysteine positions” column states the positions of cysteine residues after amino acid 30 and hence, does not include amino acids that are part of the periplasmic signal sequence which is cleaved after protein translocation. All β-lactamase enzymes except L2-1 and LUT-1 (shaded in grey), which are used as negative controls throughout this study, have one or more disulfide bonds. Both L2-1 and LUT-1 contain two or more cysteine residues, but lack disulfide bonds as they are transported to the periplasm in a folded state by the Twin-arginine translocation (Tat) system; for L2-1 Tat-dependent translocation has been experimentally confirmed ([1](#_ENREF_1)), whereas for LUT-1 this is strongly corroborated by signal peptide prediction software (SignalP 5.0 ([2](#_ENREF_2)) likelihood scores: Sec/SPI = 0.0572, Tat/SPI = 0.9312, Sec/SPII (lipoprotein) = 0.0087, other = 0.0029). The “Mob.” (mobilizable) column refers to the possibility for the β-lactamase gene to be mobilized from the chromosome; “yes” indicates that the gene of interest is located on a mobile element, while “no” refers to immobile chromosomally-encoded enzymes. The “Spectrum” column refers to the hydrolytic spectrum of each tested enzyme; tested enzymes are narrow-spectrum β-lactamases (NS), extended-spectrum β-lactamases (ESBL) or carbapenemases. The “Inh.” (inhibition) column refers to classical inhibitor susceptibility i.e., susceptibility to inhibition by clavulanic acid, tazobactam or sulbactam. Finally, the “Organism” column refers to the bacterial species that most commonly express the tested β-lactamase enzymes.

| ENZYME | CYSTEINE POSITIONS | AMBLER CLASS | MOB. | SPECTRUM | INH. | ORGANISM |
| --- | --- | --- | --- | --- | --- | --- |
| L2-1 | C82 C136 C233 | A | no | ESBL | yes | *Stenotrophomonas maltophilia* |
| LUT-1 | C54 C129 | A | no ([3](#_ENREF_3)) | NS | yes | *Pseudomonas luteola* |
| BEL-1 | C61 C231 | A | yes ([4](#_ENREF_4)) | ESBL | yes | *Pseudomonas aeruginosa* |
| CARB-2 | C72 C118 | A | yes ([5](#_ENREF_5)) | NS | yes | *Pseudomonas spp.* |
| BPS-1m | C75 C83 C129 | A | no ([6](#_ENREF_6)) | ESBL | yes | *Burkholderia pseudomallei* |
| BPS-6 | C75 C83 C129 | A | no ([7](#_ENREF_7)) | ESBL | yes | *Burkholderia pseudomallei* |
| GES-19, 20, or 26 | C63 C233 | A | yes ([8](#_ENREF_8)) | ESBLs | yes | *Enterobacteriaceae, Pseudomonas aeruginosa* |
| AIM-1 | C31 C56 C194 C199 C234 C274 | B3 | yes ([9](#_ENREF_9)) | carbapenemase | no ([10](#_ENREF_10)) | *Pseudomonas aeruginosa* |
| L1-1 | C239 C265 | B3 | no ([10](#_ENREF_10)) | carbapenemase | no ([10](#_ENREF_10)) | *Stenotrophomonas maltophilia* |
| POM-1 | C237 C265 | B3 | no ([11](#_ENREF_11)) | carbapenemase | no ([10](#_ENREF_10)) | *Pseudomonas otitidis* |
| SMB-1 | C180 C 185 C226 C260 | B3 | yes ([12](#_ENREF_12)) | carbapenemase | no ([10](#_ENREF_10)) | *Serratia spp.* |
| OXA-50 | C208 C211 | D | no ([13](#_ENREF_13)) | NS | no ([13](#_ENREF_13)) | *Pseudomonas spp.* |

**Supplementary Table 2.** Bacterial strains used in this study. All listed isolates are clinical strains. “FNRCAR” refers to the French National Reference Centre for Antibiotic Resistance in Le Kremlin-Bicêtre, France, and “CDC AR Isolate bank” refers to the Centers for Disease Control and Prevention Antibiotic Resistance Isolate Bank in Atlanta, GA, USA.

| **NAME** | **DESCRIPTION** | **SOURCE** |
| --- | --- | --- |
| ***Escherichia coli*** | | |
| DH5α | F^–^ *end*A1 *gln*V44 *thi*-1 *rec*A1 *rel*A1 *gyr*A96 *deo*R *nup*G *pur*B20 φ80d*lacZ*∆M15 ∆(*lac*ZYA*-arg*F*)*U169 *hsd*R17(r_K_^–^m_K_^+^) λ^–^ | ([14](#_ENREF_14)) |
| DH5αλpir | λ*pir* | ([15](#_ENREF_15)) |
| CC118λpir | *ara*D Δ(*ara*, *leu*) Δ*lac*Z74 *pho*A20 *gal*K *thi*-1 *rsp*E *rpo*B *arg*E *rec*A1 λ*pir* | ([16](#_ENREF_16)) |
| HB101 | supE44 hsdS20 recA13 ara-14 proA2 lacY1 galK2 rpsL20 xyl-5 mtl-1 | ([17](#_ENREF_17)) |
| MC1000 | *ara*D139 ∆(*ara, leu*)7697 ∆*lac*X74 *gal*U *gal*K *str*A | ([18](#_ENREF_18)) |
| MC1000 *dsbA* | *dsbA::aphA*, Kan^R^ | ([19](#_ENREF_19)) |
| MC1000 *dsbA* *att*Tn*7*::P*tac-dsbA* | *dsbA::aphA* *att*Tn*7*::*dsbA*, Kan^R^ | ([20](#_ENREF_20)) |
| **Clinical isolates** | | |
| *Pseudomonas aeruginosa* PAO1 | wild-type prototroph | ([21](#_ENREF_21)) |
| *Pseudomonas aeruginosa* PA14 | wild-type prototroph | ([22](#_ENREF_22)) |
| *Pseudomonas aeruginosa* PA14 *att*Tn*7::accC* | *att*Tn*7::accC*, Gent^R^ | This study |
| *Pseudomonas aeruginosa* G4R7 | *bla*_AIM-1_ | FNRCAR |
| *Pseudomonas aeruginosa* G4R7 *dsbA1* | *dsbA1* *bla*_AIM-1_ | This study |
| *Pseudomonas aeruginosa* G6R7 | *bla*_AIM-1_ | FNRCAR |
| *Pseudomonas aeruginosa* G6R7 *dsbA1* | *dsbA1* *bla*_AIM-1_ | This study |
| *Pseudomonas aeruginosa* CDC #769 | *bla*_GES-19_ *bla*_GES-26_ | CDC AR Isolate Bank |
| *Pseudomonas aeruginosa* CDC #769 *dsbA1* | *dsbA1 bla*_GES-19_ *bla*_GES-26_ | This study |
| *Pseudomonas aeruginosa* CDC #769 *dsbA1 att*Tn*7::accC msfgfp dsbA1* | *dsbA1 bla*_GES-19_ *bla*_GES-26_ *att*Tn*7::accC* *msfgfp dsbA1*, Gent^R^ | This study |
| *Pseudomonas aeruginosa* CDC #773 | *bla*_GES-19_ *bla*_GES-20_ | CDC AR Isolate Bank |
| *Pseudomonas aeruginosa* CDC #773 *dsbA1* | *dsbA1 bla*_GES-19_ *bla*_GES-20_ | This study |
| *Stenotrophomonas maltophilia* AMM | *bla*_L2-1_ *bla*_L1-1_ | ([23](#_ENREF_23)) |
| *Stenotrophomonas maltophilia* AMM *dsbA dsbL* | *dsbA* *dsbL* *bla*_L2-1_ *bla*_L1-1_ | This study |
| *Stenotrophomonas maltophilia* AMM *att*Tn*7::accC msfgfp* | *bla*_L2-1_ *bla*_L1-1_  *att*Tn*7::accC msfgfp,* Gent^R^ | This study |
| *Stenotrophomonas maltophilia* AMM *dsbA dsbL att*Tn*7::accC msfgfp* | *dsbA* *dsbL* *bla*_L2-1_ *bla*_L1-1_ *att*Tn*7::accC msfgfp*, Gent^R^ | This study |
| *Stenotrophomonas maltophilia* AMM *dsbA dsbL att*Tn7*::accC msfgfp dsbA1* | *dsbA* *dsbL* *bla*_L2-1_ *bla*_L1-1_ *att*Tn*7::accC* *msfgfp* *dsbA1*, Gent^R^ | This study |
| *Stenotrophomonas maltophilia* GUE | *bla*_L2-1_ *bla*_L1-1_ | ([23](#_ENREF_23)) |
| *Stenotrophomonas maltophilia* GUE  *dsbA dsbL* | *dsbA* *dsbL* *bla*_L2-1_ *bla*_L1-1_ | This study |

**Supplementary Table 3.** Plasmids used in this study.

| NAME | DESCRIPTION | SOURCE |
| --- | --- | --- |
| pDM1 | pDM1 vector (GenBank MN128719), p15A *ori*, P*tac* promoter, MCS, Tet^R^ | Mavridou lab stock |
| pDM1-*bla*_L2-1_ | *bla*_L2-1_ cloned into pDM1, Tet^R^ | ([20](#_ENREF_20)) |
| pDM1-*bla*_LUT-1_ | *bla*_LUT-1_ cloned into pDM1, Tet^R^ | This study |
| pDM1-*bla*_BEL-1_ | *bla*_BEL-1_ cloned into pDM1, Tet^R^ | This study |
| pDM1-*bla*_CARB-2_ | *bla*_CARB-2_ cloned into pDM1, Tet^R^ | This study |
| pDM1-*bla*_BPS-1m_ | *bla*_BPS-1m_ cloned into pDM1, Tet^R^ | This study |
| pDM1-*bla*_BPS-6_ | *bla*_BPS-6_ cloned into pDM1, Tet^R^ | This study |
| pDM1-*bla*_AIM-1_ | *bla*_AIM-1_ cloned into pDM1, Tet^R^ | This study |
| pDM1-*bla*_POM-1_ | *bla*_POM-1_ cloned into pDM1, Tet^R^ | This study |
| pDM1-*bla*_SMB-1_ | *bla*_SMB-1_ cloned into pDM1, Tet^R^ | This study |
| pDM1-*bla*_OXA-50_ | *bla*_OXA-50_ cloned into pDM1, Tet^R^ | This study |
| pDM1-*bla*_BEL-1_-StrepII | *bla*_BEL-1_ encoding BEL-1 with a C-terminal StrepII tag cloned into pDM1, Tet^R^ | This study |
| pDM1-*bla*_CARB-2_-StrepII | *bla*_CARB-2_ encoding CARB-2 with a C-terminal StrepII tag cloned into pDM1, Tet^R^ | This study |
| pDM1-*bla*_BPS-1m_-StrepII | *bla*_BPS-1m_ encoding BPS-1m with a C-terminal StrepII tag cloned into pDM1, Tet^R^ | This study |
| pDM1-*bla*_AIM-1_-StrepII | *bla*_AIM-1_ encoding AIM-1 with a C-terminal StrepII tag cloned into pDM1, Tet^R^ | This study |
| pDM1-*bla*_L2-1_-StrepII | *bla*_L2-1_ encoding L2-1 with a C-terminal StrepII tag cloned into pDM1, Tet^R^ | ([20](#_ENREF_20)) |
| pDM1-*bla*_POM-1_-StrepII | *bla*_POM-1_ encoding POM-1 with a C-terminal StrepII tag cloned into pDM1, Tet^R^ | This study |
| pDM1-*bla*_SMB-1_-StrepII | *bla*_SMB-1_ encoding SMB-1 with a C-terminal StrepII tag cloned into pDM1, Tet^R^ | This study |
| pDM1-*bla*_OXA-50_-StrepII | *bla*_OXA-50_ encoding OXA-50 with a C-terminal StrepII tag cloned into pDM1, Tet^R^ | This study |
| pKNG101 | Gene replacement suicide vector, *ori*R6K, *oriT*RK2, *sacB*, Str^R^ | ([24](#_ENREF_24)) |
| pKNG102 | Gene replacement suicide vector, *ori*R6K, *oriT*RK2, *sacB*, Tet^R^ | Bernal lab stock |
| pKNG101-*dsbA1* | PCR fragment containing the regions upstream and downstream *P. aeruginosa dsbA1* cloned in pKNG101; when inserted into the chromosome, the strain is a merodiploid for *dsbA1* mutant, Str^R^ | ([20](#_ENREF_20)) |
| pKNG102-*dsbA1*-769 | PCR fragment containing the regions upstream and downstream *P. aeruginosa* CDC #769 (Supplementary Table 2) *dsbA1* cloned in pKNG102; when inserted into the chromosome, the strain is a merodiploid for *dsbA1* mutant, Tet^R^ | This study |
| pKNG102-*dsbA1*-773 | PCR fragment containing the regions upstream and downstream *P. aeruginosa* CDC #773 (Supplementary Table 2) *dsbA1* cloned in pKNG102; when inserted into the chromosome, the strain is a merodiploid for *dsbA1* mutant, Tet^R^ | This study |
| pKNG101-*dsbA dsbL*-AMM | PCR fragment containing the regions upstream and downstream *S. maltophilia* AMM *dsbA* and *dsbL* genes cloned in pKNG101; when inserted into the chromosome, the strain is a merodiploid for *dsbA dsbL* mutant, Str^R^ | This study |
| pKNG101-*dsbA dsbL*-GUE | PCR fragment containing the regions upstream and downstream *S. maltophilia* GUE *dsbA* and *dsbL* genes cloned in pKNG101; when inserted into the chromosome, the strain is a merodiploid for *dsbA dsbL* mutant, Str^R^ | This study |
| pRK600 | Helper plasmid, ColE1 ori, mobRK2, traRK2, Cam^R^ | ([25](#_ENREF_25)) |
| pTn*7*-M | Mini-Tn*7* delivery transposon vector containing the Tn*7* flanking regions and a Gent^R^ marker, R6K *ori*, Kan^R^, Gent^R^ | ([26](#_ENREF_26)) |
| pBG42 | Mini-Tn*7* delivery transposon vector containing the Tn*7* flanking regions, a Gent^R^ marker and *msfgfp,* R6K *ori*, Kan^R^, Gent^R^ | ([26](#_ENREF_26)) |
| pBG42-PAO1*dsbA1* | *dsbA1* encoding DsbA1 from *P. aeruginosa* PAO1 cloned into pBG42, Kan^R^, Gent^R^ | This study |
| pTNS2 | Helper plasmid, R6K *ori*; encodes the TnsABC+D specific transposition pathway, Amp^R^ | ([27](#_ENREF_27)) |
| pMK-RQ *carb-2* | GeneArt® cloning vector containing *carb-2*, ColE1 *ori*, (template for *carb-2*), Kan^R^ | This study |
| pMK-RQ *bps-1m* | GeneArt® cloning vector containing *bps-1m*, ColE1 *ori*, (template for *bps-1m*), Kan^R^ | This study |
| pMK-RQ *bps-6* | GeneArt® cloning vector containing *bps-6*, ColE1 *ori*, (template for *bps-6*), Kan^R^ | This study |
| pMK-RQ *smb-1* | GeneArt® cloning vector containing *smb-1*, ColE1 *ori*, (template for *smb-1*), Kan^R^ | This study |

**Supplementary Table 4.** Oligonucleotide primers used in this study. The “Brief description” column provides basic information on the primer design (restriction enzyme used for cloning, encoded protein or gene replaced by antibiotic resistance cassette, forward or reverse orientation of the primer (F or R); SQ stands for sequencing primers).

| **NUMBER** | **BRIEF DESCRIPTION** | **SEQUENCE (5ˊ-3ˊ)** |
| --- | --- | --- |
| P1 | SacI.LUT-1.F | ctggagctcaatgtcatcctgaaccgtcga |
| P2 | PstI.LUT-1.R | cagctgcagtcagcctgtcacccattcag |
| P3 | SacI.BEL-1.F | ctggagctcaaactgctctacccgttattgc |
| P4 | PstI.BEL-1.R | cagctgcagtcagtgaacatattgacgtgc |
| P5 | SacI.CARB-2.F | ctggagctcaagtttttattggcattttcgc |
| P6 | KpnI.CARB-2.R | cagggtacctcagcgcgactgtgatgta |
| P7 | SacI.BPS-1m.F | ctggagctcaatcattctccgttgcgccgctc |
| P8 | XmaI.BPS-1m.R | caacccgggtcaggcgaacgcccgcgcg |
| P9 | SacI.AIM-1.F | ctggagctcaaacgtcgcttcaccctgg |
| P10 | KpnI.AIM-1.R | ctgggtacctcaaggccgcgcgccgctg |
| P11 | SacI.POM-1.F | ctggagctccgtaccctgaccctcg |
| P12 | KpnI.POM-1.R | cagggtaccttatgcgtcatcagagacctc |
| P13 | NdeI.SMB-1.F | cagctccatatgaaaatcatcgcttccctgatcc |
| P14 | XmaI.SMB-1.R | ctgcccgggtcagcgtttctcgctggcca |
| P15 | SacI.OXA-50.F | ctggagctccgccctctcttcagtg |
| P16 | KpnI.OXA-50.R | cagggtacctcagggcagtatcccgagag |
| P17 | PstI.StrepII.BEL-1.R | cagctgcagttatttttcaaattgcggatggctccaagcgctcccgtgaacatattgacgtgctaac |
| P18 | KpnI.StrepII.CARB-2.R | cagggtaccttatttttcaaattgcggatggctccaagcgctcccgcgcgactgtgatgtataa |
| P19 | XmaI.StrepII.BPS-1m.R | ctgcccgggctatttttcaaattgcggatggctccaagcgctcccggcgaacgcccgcgcggcg |
| P20 | KpnI.StrepII.AIM-1.R | cagggtaccttatttttcaaattgcggatggctccaagcgctcccaggccgcgcgccgctggag |
| P21 | KpnI.StrepII.POM-1.R | cagggtaccttatttttcaaattgcggatggctccaagcgctcccgccgcgctgcttc |
| P22 | XmaI.StrepII.SMB-1.R | ctgcccgggctatttttcaaattgcggatggctccaagcgctcccgcgtttctcgctggccag |
| P23 | KpnI.StrepII.OXA-50.R | cagggtaccttatttttcaaattgcggatggctccaagcgctcccgggcagtatcccgagagcc |
| P24 | SQ.dsbA1.Paeruginosa.F | tacctgctcaagcagatgcatg |
| P25 | SQ.dsbA1.Paeruginosa.R | ggtgttcatgtcgcccatca |
| P26 | SQ.dsbAdsbL.Smaltophilia.F | atggtgccgttcgtgcaga |
| P27 | SQ.dsbAdsbL.Smaltophilia.R | acagcacctgcatttccgg |
| P28 | XbaI.dsbA1.F | ggttcctctagagcctacttcgccagccagaa |
| P29 | pKNG101-dsbA1.body.R | ctacttcttgttacgcatcgttcactc |
| P30 | pKNG101-dsbA1.body.F | atgcgtaacaagaagtaggcaaggtga |
| P31 | BamHI.dsbA1.R | aattaaggatcctcatcactaccaccagcgcg |
| P32 | XbaI.dsbAdsbL.F | ggttcctctagatcttctggtacagcacctgcatttccg |
| P33 | pKNG102-dsbAdsbL.body.R | tgcgtgtcgatgaggttggctcactga |
| P34 | pKNG102-dsbAdsbL.body.F | tctcttggatcagtgagccaacctcat |
| P35 | BamHI.dsbAdsbL.R | aattaaggatcctcgctggaggtggatttcagcaagacc |
| P36 | pBG42-vector.F | gaattcgagctcggtaccc |
| P37 | pBG42-vector.R | tagaaaacctccttagcatgattaagatg |
| P38 | PAO1dsbA1-insert.F | catgctaaggaggttttctaatgcgtaacctgattctcacc |
| P39 | PAO1dsbA1-insert.R | gtaccgagctcgaattcctacttcttggccgctgc |
| P40 | HindIII.PEM7-msfgfp.F | cacaaagctttgttgacaattaatcatcggcatagtatatcggcatagtataatacgacaaggtgaggaactaaaccaggaggaaaaacatatgcgtaaaggtgaagaactgttcac |
| P41 | msfgfp.BamHI.R | cacaggatccttatttgtagagttcatccatgccg |
| P42 | SQ.pBG42-PAO1dsbA1.F | ccgctgcgttcggtc |
| P43 | SQ.pBG42-PAO1dsbA1.R | ccaagactagtcgccagg |
| P44 | SQ.Tn7.Paeruginosa.F | gtcgaagccgagctggtg |
| P45 | SQ.Tn7.Paeruginosa.R | gatcgccaagggtgcctg |
| P46 | SQ.Tn7.Smaltophilia.F | gtcgatgccgcccaagaag |
| P47 | SQ.Tn7.Smaltophilia.R | gatggcaccttccatgagaac |

**Supplementary Table 5.** Sources of genomic DNA used for amplification of β-lactamase genes used in this study. CRBIP stands for Centre de Ressources Biologiques de l’Institut Pasteur, France and FNRCAR refers to the French National Reference Centre for Antibiotic Resistance in Le Kremlin-Bicêtre, France.

| **STRAIN** | **GENE** | **SOURCE** |
| --- | --- | --- |
| *Pseudomonas aeruginosa* 51170 | *bla*_BEL-1_ | ([4](#_ENREF_4)) |
| *Pseudomonas luteola* CIP 102067 | *bla*_LUT-1_ | CRBIP |
| *Pseudomonas aeruginosa* G4R7 | *bla*_AIM-1_ | FNRCAR |
| *Pseudomonas otitidis* CIP 109236T | *bla*_POM-1_ | CRBIP |
| *Pseudomonas aeruginosa* PAO1 LA | *bla*_OXA-50_ | ([21](#_ENREF_21)) |

**SUPPLEMENTARY REFERENCES**

1. Pradel N, Delmas J, Wu LF, Santini CL, Bonnet R. Sec- and Tat-dependent translocation of b-lactamases across the *Escherichia coli* inner membrane. Antimicrob. Agents Chemother. 2009;53(1):242-8.

2. Almagro Armenteros JJ, Tsirigos KD, Sonderby CK, Petersen TN, Winther O, Brunak S, et al. SignalP 5.0 improves signal peptide predictions using deep neural networks. Nat. Biotechnol. 2019;37(4):420-3.

3. Doublet B, Robin F, Casin I, Fabre L, Le Fleche A, Bonnet R, et al. Molecular and biochemical characterization of the natural chromosome-encoded class A b-lactamase from *Pseudomonas luteola*. Antimicrob. Agents Chemother. 2010;54(1):45-51.

4. Poirel L, Brinas L, Verlinde A, Ide L, Nordmann P. BEL-1, a novel clavulanic acid-inhibited extended-spectrum b-lactamase, and the class 1 integron In120 in *Pseudomonas aeruginosa*. Antimicrob. Agents Chemother. 2005;49(9):3743-8.

5. Bert F, Branger C, Lambert-Zechovsky N. Identification of PSE and OXA b-lactamase genes in *Pseudomonas aeruginosa* using PCR-restriction fragment length polymorphism. J. Antimicrob. Chemother. 2002;50(1):11-8.

6. Ho PL, Cheung TKM, Yam WC, Yuen KY. Characterization of a laboratory-generated variant of BPS b-lactamase from *Burkholderia pseudomallei* that hydrolyses ceftazidime. J. Antimicrob. Chemother. 2002;50(5):723-6.

7. Tribuddharat C, Moore RA, Baker P, Woods DE. *Burkholderia pseudomallei* class a b-lactamase mutations that confer selective resistance against ceftazidime or clavulanic acid inhibition. Antimicrob. Agents Chemother. 2003;47(7):2082-7.

8. Yoon EJ, Jeong SH. Mobile carbapenemase genes in *Pseudomonas aeruginosa*. Front. Microbiol. 2021;12:614058.

9. Yong D, Toleman MA, Bell J, Ritchie B, Pratt R, Ryley H, et al. Genetic and biochemical characterization of an acquired subgroup B3 metallo-b-lactamase gene, *bla*_AIM-1_, and its unique genetic context in *Pseudomonas aeruginosa* from Australia. Antimicrob. Agents Chemother. 2012;56(12):6154-9.

10. Tooke CL, Hinchliffe P, Bragginton EC, Colenso CK, Hirvonen VHA, Takebayashi Y, et al. b-Lactamases and b-lactamase inhibitors in the 21st century. J. Mol. Biol. 2019;431(18):3472-500.

11. Thaller MC, Borgianni L, Di Lallo G, Chong Y, Lee K, Dajcs J, et al. Metallo-b-lactamase production by *Pseudomonas otitidis*: a species-related trait. Antimicrob. Agents Chemother. 2011;55(1):118-23.

12. Wachino J, Yoshida H, Yamane K, Suzuki S, Matsui M, Yamagishi T, et al. SMB-1, a novel subclass B3 metallo-b-lactamase, associated with ISCR1 and a class 1 integron, from a carbapenem-resistant *Serratia marcescens* clinical isolate. Antimicrob. Agents Chemother. 2011;55(11):5143-9.

13. Girlich D, Naas T, Nordmann P. Biochemical characterization of the naturally occurring oxacillinase OXA-50 of *Pseudomonas aeruginosa*. Antimicrob. Agents Chemother. 2004;48(6):2043-8.

14. Hanahan D. In: Glover DM, editor. DNA Cloning: A Practical Approach. 1: IRL Press, McLean, Virginia; 1985. p. 109.

15. Martinez-Garcia E, de Lorenzo V. Engineering multiple genomic deletions in Gram-negative bacteria: analysis of the multi-resistant antibiotic profile of *Pseudomonas putida* KT2440. Env. Microbiol. 2011;13(10):2702-16.

16. Herrero M, de Lorenzo V, Timmis KN. Transposon vectors containing non-antibiotic resistance selection markers for cloning and stable chromosomal insertion of foreign genes in Gram-negative bacteria. J. Bacteriol. 1990;172(11):6557-67.

17. Boyer HW, Roulland-Dussoix D. A complementation analysis of the restriction and modification of DNA in *Escherichia coli*. J. Mol. Biol. 1969;41(3):459-72.

18. Casadaban MJ, Cohen SN. Analysis of gene control signals by DNA fusion and cloning in *Escherichia coli*. J. Mol. Biol. 1980;138(2):179-207.

19. Kadokura H, Tian H, Zander T, Bardwell JC, Beckwith J. Snapshots of DsbA in action: detection of proteins in the process of oxidative folding. Science. 2004;303(5657):534-7.

20. Furniss RCD, Kaderabkova N, Barker D, Bernal P, Maslova E, Antwi AAA, et al. Breaking antimicrobial resistance by disrupting extracytoplasmic protein folding. eLife. 2022;11.

21. Holloway BW. Genetics of *Pseudomonas*. Bacteriol. Rev. 1969;33(3):419-43.

22. Rahme LG, Stevens EJ, Wolfort SF, Shao J, Tompkins RG, Ausubel FM. Common virulence factors for bacterial pathogenicity in plants and animals. Science. 1995;268(5219):1899-902.

23. Emeraud C, Escaut L, Boucly A, Fortineau N, Bonnin RA, Naas T, et al. Aztreonam plus clavulanate, tazobactam, or avibactam for treatment of infections caused by metallo-b-lactamase-producing Gram-negative bacteria. Antimicrob. Agents Chemother. 2019;63(5).

24. Kaniga K, Delor I, Cornelis GR. A wide-host-range suicide vector for improving reverse genetics in Gram-negative bacteria: inactivation of the *blaA* gene of *Yersinia enterocolitica*. Gene. 1991;109(1):137-41.

25. Kessler B, Delorenzo V, Timmis KN. A general system to integrate *lacZ* fusions into the chromosomes of Gram-negative eubacteria: regulation of the *Pm* Promoter of the *TOL* plasmid studied with all controlling elements in monocopy. Mol. Gen. Genet. 1992;233(1-2):293-301.

26. Zobel S, Benedetti I, Eisenbach L, de Lorenzo V, Wierckx N, Blank LM. Tn*7*-based device for calibrated heterologous gene expression in *Pseudomonas putida*. ACS Synth. Biol. 2015;4(12):1341-51.

27. Choi KH, Gaynor JB, White KG, Lopez C, Bosio CM, Karkhoff-Schweizer RR, et al. A Tn*7*-based broad-range bacterial cloning and expression system. Nat. Methods. 2005;2(6):443-8.
